# A pectin lyase-like protein from *Verticillium dahliae* activates immunity in eggplant through translation regulation

**DOI:** 10.1093/hr/uhaf050

**Published:** 2025-02-18

**Authors:** Chi Li, Shuaifei Cui, Yuzhen Li, Xiaoshi Liu, Wei Yan, Chengluo Zhu, Baojuan Sun, Chengwei Yang, Tao Li, Jianbin Lai

**Affiliations:** Guangdong Provincial Key Laboratory of Biotechnology for Plant Development, School of Life Sciences, South China Normal University, 510631 Guangzhou, China; Guangdong Provincial Key Laboratory of Biotechnology for Plant Development, School of Life Sciences, South China Normal University, 510631 Guangzhou, China; Guangdong Provincial Key Laboratory of Biotechnology for Plant Development, School of Life Sciences, South China Normal University, 510631 Guangzhou, China; Guangdong Provincial Key Laboratory of Biotechnology for Plant Development, School of Life Sciences, South China Normal University, 510631 Guangzhou, China; Guangdong Provincial Key Laboratory of Biotechnology for Plant Development, School of Life Sciences, South China Normal University, 510631 Guangzhou, China; Guangdong Provincial Key Laboratory of Biotechnology for Plant Development, School of Life Sciences, South China Normal University, 510631 Guangzhou, China; Guangdong Academy of Agricultural Sciences, Guangdong Key Laboratory for New Technology Research of Vegetables, Vegetable Research Institute, Guangzhou 510640, China; Guangdong Provincial Key Laboratory of Biotechnology for Plant Development, School of Life Sciences, South China Normal University, 510631 Guangzhou, China; Guangdong Academy of Agricultural Sciences, Guangdong Key Laboratory for New Technology Research of Vegetables, Vegetable Research Institute, Guangzhou 510640, China; Guangdong Provincial Key Laboratory of Biotechnology for Plant Development, School of Life Sciences, South China Normal University, 510631 Guangzhou, China

Dear Editor,

Eggplant (*Solanum melongena* L.) is a widely cultivated vegetable crop, but it is frequently susceptible to various pathogens, leading to significant yield losses. Verticillium wilt, caused by *Verticillium dahliae* is an important disease affecting the production of a wide range of host plants. Infection with *V. dahliae* via the vascular system disrupts uptake of water and nutrients, resulting in symptoms such as wilting, chlorosis, necrosis, vein clearing, and stunting [1]. Given that eggplant is highly susceptible to *V. dahliae* and that verticillium wilt poses a significant threat to eggplant production, it is essential to elucidate the mechanisms underlying the interaction between *V. dahliae* and eggplant. During infection, *V. dahliae* secretes a significant array of carbohydrate-active enzymes, including pectin lyases, which may facilitate the destruction of plant cell walls to enhance invasion by *V. dahliae* [2]; for instance, the verticillium polysaccharide deacetylase contributes to efficient infection [3]. However, the roles of fungal carbohydrate-active enzymes in the activation of eggplant immunity remain unclear.

**Figure 1:**
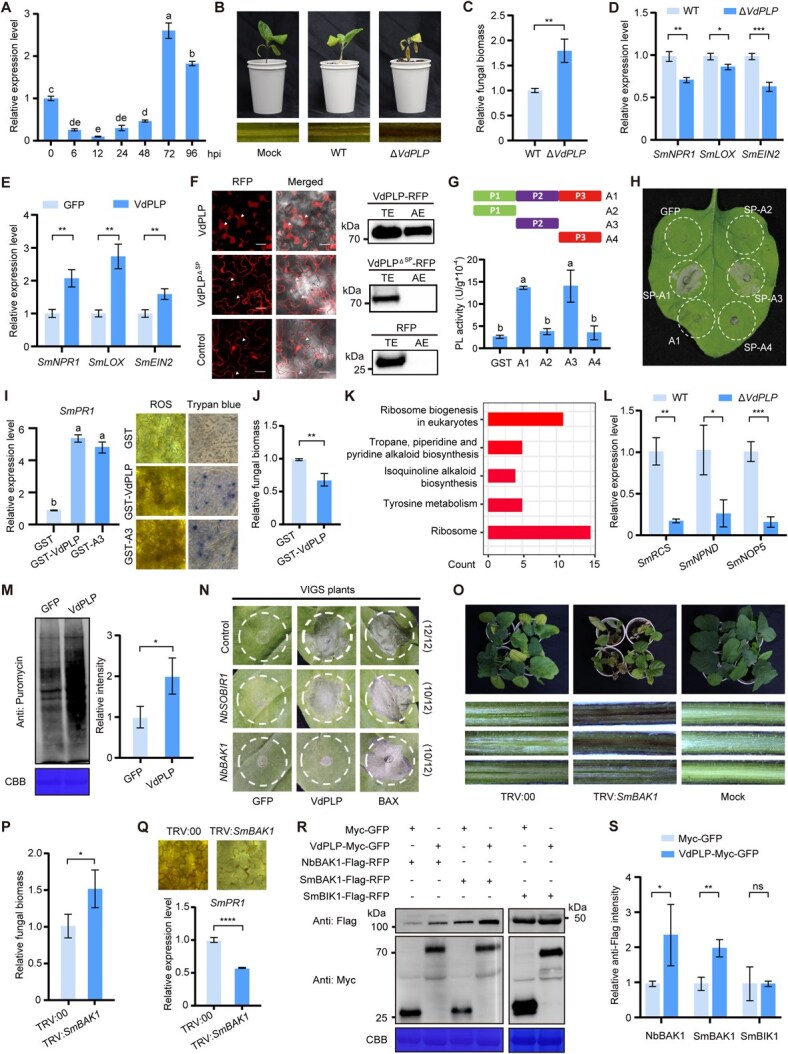
The role of VdPLP in the infection process of eggplant by *V. dahliae*. (A) Expression pattern analysis of *VdPLP* during *V. dahliae* infection of eggplant using RT–qPCR. (B, C) Symptoms of eggplant infected by wild-type and Δ*VdPLP* strains of *V. dahliae*. Representative symptoms at 14 days post-inoculation are shown in (B) and relative fungal biomass determined by qPCR is presented in (C). (D) Relative expression analysis of defense-related genes in leaves of eggplants inoculated with either wild-type or Δ*VdPLP* strains of *V. dahliae* for 72 hours using RT–qPCR. (E) Relative expression analysis of defense-related genes in eggplant leaves expressing either GFP or VdPLP-GFP for 72 hours using RT–qPCR. (F) Subcellular localization of VdPLP–RFP with or without SP. Representative RFP signals in *N. benthamiana* leaves upon plasmolysis are shown in the left graph. Bars = 20 μm. Anti-RFP immunoblots utilized to detect proteins in apoplast extracts (AE) and total extracts (TE) from *N. benthamiana* leaves are shown in the right graph. (G) Determination of pectin lyase activity of different forms of purified VdPLP. (H) Cell death induced by different forms derived from VdPLP in *N. benthamiana*. (I) Impact of treating eggplants with purified VdPLP. Relative expression of *SmPR1* (24 hours, RT–qPCR), ROS level (24 hours, diaminobenzidine staining), and cell death (72 hours, trypan blue staining). (J) Effect of purified VdPLP on *V. dahliae* infection quantified by qPCR. (K) KEGG pathway analysis using upregulated eggplant genes in the sample with wild-type *V. dahliae* infection, compared with that with Δ*VdPLP*. Eggplant hypocotyls were harvested 72 hours post-inoculation for RNA-seq. (L) Verification of differentially expressed genes associated with ribosome biosynthesis using RT–qPCR. (M) Impact of VdPLP on plant protein synthesis. Leaves expressing VdPLP–GFP or GFP were labeled with 50 μM puromycin for 1 hour and subjected to immunoblotting using an anti-puromycin antibody. (N) Role of BAK1 in VdPLP-induced cell death. VIGS was employed to silence *NbSOBIR1* or *NbBAK1* in leaves of *N. benthamiana* for 2 weeks, and then VdPLP was expressed for 7 days. (O, P) Impact of *SmBAK1* knockdown on eggplant resistance to *V. dahliae*. Symptoms 14 days post-inoculation are shown in (O) and relative fungal biomass determined by qPCR in (P). (Q) Impact of purified VdPLP on *SmBAK1*-silenced eggplants. Levels of ROS and *SmPR1* expression were detected 24 hours after treatment. (R, S) Impact of VdPLP on protein accumulation of BAK1. SmBIK1 was utilized as a control. Representative immunoblots are shown in (R) and relative protein intensity is shown in (S). All results are representative of three biologically independent experiments. Quantitative data are mean ± standard deviation from three replicates. Significance analysis in (A), (G), and (I) was performed using one-way ANOVA followed by Tukey’s multiple comparison test (*P* < 0.05); significance analysis in (C), (D), (E), (J), (L), (M), (P), (Q), and (S) was performed using Student’s *t*-test (^*^P < 0.05; ^**^P < 0.01; ^***^P < 0.001; ^****^P < 0.0001; ns, not significant).

Based on bioinformatics analysis, here we identified a pectin lyase-like protein with a potential signal peptide (SP) from *V. dahliae*, designated VdPLP. The RT–qPCR result indicated a significant increase in the transcript level of *VdPLP* at 72 hours post-inoculation with *V. dahliae* on eggplant (Fig. 1A), supporting its potential function during infection. Inoculation with the wild-type *V. dahliae* induced mild symptoms in eggplant plants, including wilting, etiolation, leaf abscission, and vascular discoloration. Surprisingly, these disease symptoms were significantly enhanced by the mutant strain with depletion of *VdPLP* (Δ*VdPLP*) (Fig. 1B). The data on fungal biomass and defense gene expression supported the idea that the mutation of *VdPLP* attenuated eggplant immunity and facilitated *V. dahliae* infection (Fig. 1C and D). Consistently, overexpression of VdPLP promoted the expression of defense-related genes in eggplant leaves (Fig. 1E), suggesting that this fungal protein is involved in activation of eggplant immunity.

Therefore, we further investigated the molecular mechanism by which VdPLP functions in plants. Our confocal microscopy and apoplastic fluid immunoblot data confirmed the localization of VdPLP in the apoplastic region of *Nicotiana benthamiana* leaves (Fig. 1F), implying that it may be secreted from *V. dahliae* cells. *In vitro* assay data supported the idea that VdPLP exhibited pectin lyase activity, with the central region (A3) being sufficient to sustain its enzymatic function (Fig. 1G). Removal of the SP abolished the phenotype induced by VdPLP, whereas fusion of the SP with the central region (A3) resulted in cell death similar to full-length VdPLP (Fig. 1H). Furthermore, the purified recombinant GST-tagged VdPLP from *E. coli* was utilized for treating eggplants. In comparison with the GST control, treatment with either the full-length (without SP) or A3 version of VdPLP resulted in upregulation of the defense gene *SmPR1*, increased accumulation of reactive oxygen species (ROS), and enhanced cell death (Fig. 1I). Treatment with purified VdPLP also reduced the accumulation of *V. dahliae* in infected eggplants (Fig. 1J). These findings suggested that VdPLP functions as a pectin lyase, facilitating immunity activation in apoplastic regions.

To elucidate the signaling pathways in eggplant that are regulated by VdPLP during infection, we conducted a transcriptomic analysis using eggplant samples collected 72 hours post-inoculation with either the wild-type or Δ*VdPLP* variant of *V. dahliae* (the RNA-seq data were deposited in BioProject under accession number PRJNA1212702). Notably, KEGG analysis of upregulated genes in samples infected with wild-type *V. dahliae* revealed significant enrichment in ribosome biogenesis pathways (Fig. 1K). Verification through RT–qPCR indicated that the expression levels of genes related to ribosome biosynthesis in eggplant were reduced in response to Δ*VdPLP* (Fig. 1L). We hypothesized that VdPLP may upregulate host translation during *V. dahliae* infection, and therefore measured the global levels of protein synthesis in *N. benthamiana* leaves with and without VdPLP. The result with puromycin labeling [4] revealed that the level of newly synthesized proteins was significantly elevated in the presence of VdPLP (Fig. 1M), suggesting that it enhances host protein synthesis.

Therefore, the synthesis of certain host immune factors may also be upregulated in response to VdPLP, and thus knockdown of these host genes could alleviate the cell death induced by VdPLP. We initiated this detection in *N. benthamiana* leaves by downregulating critical immune genes, such as *NbSOBIR1* and *NbBAK1* [5], through virus-induced gene silencing (VIGS). Notably, the knockdown of *NbBAK1*, rather than *NbSOBIR1*, suppressed VdPLP-induced cell death (Fig. 1N). Consistently, following inoculation with *V. dahliae*, more severe symptoms and higher fungal biomass were detected in the *SmBAK1*-silenced eggplant plants compared with the control (Fig. 1O and P). Consistently, the impact of purified VdPLP on ROS accumulation and *SmPR1* expression was attenuated in the *SmBAK1*-silenced eggplants (Fig. 1Q), supporting the role of SmBAK1 in this process. We further measured the protein levels of BAK1 with or without VdPLP, using samples with similar *BAK1* transcript levels. When co-expressed with VdPLP, the protein levels of both SmBAK1 and NbBAK1 were elevated, but the protein level of another immune-related protein, SmBIK1, did not show apparent changes (Fig. 1R and S). This observation supports the notion that VdPLP enhances the translation of the host immune factor BAK1.

As a model, VdPLP is secreted by *V. dahliae* to facilitate the degradation of host cell walls for infection, but this process leads to the upregulation of ribosome biosynthesis pathways and the consequent increased accumulation of certain immune proteins, such as BAK1, which triggers immunity activation to suppress the infection in eggplant. It remains a possibility that VdPLP may serve as a pathogen-associated molecular pattern, but another plausible mechanism is that it may target the plant cell wall to generate damage-associated molecular patterns for activating downstream immune pathways. Characterization of the residues contributing to the enzymatic activity of VdPLP will enhance our understanding of its working mechanism. Nevertheless, further investigation is required to clarify the mechanism linking cell wall integrity to translation modulation. Based on our current mechanism underlying pathogen–host competition, we propose that novel strategies could be developed to bolster resistance against verticillium wilt in eggplant and other host plants.

## Data Availability

All the data will be shared on request to the corresponding authors.
